# Supplementary material for: DNA circles promote yeast ageing in part through stimulating the reorganization of nuclear pore complexes
Source: eLife. 2022 Apr 4;11:e71196. doi: 10.7554/eLife.71196 (PMC9020822; doi:10.7554/eLife.71196)
Supplement: Transparent reporting form [file elife-71196-transrepform1.docx]

***eLife’s* transparent reporting form**

We encourage authors to provide detailed information *within their submission* to facilitate the interpretation and replication of experiments. Authors can upload supporting documentation to indicate the use of appropriate reporting guidelines for health-related research (see [EQUATOR Network](about:blank)), life science research (see the BioSharing Information Resource), or the [ARRIVE guidelines](about:blank) for reporting work involving animal research. Where applicable, authors should refer to any relevant reporting standards documents in this form.

If you have any questions, please consult our Journal Policies and/or contact us: [editorial@elifesciences.org](about:blank).

**Sample-size estimation**

- You should state whether an appropriate sample size was computed when the study was being designed
- You should state the statistical method of sample size computation and any required assumptions
- If no explicit power analysis was used, you should describe how you decided what sample (replicate) size (number) to use

Please outline where this information can be found within the submission (e.g., sections or figure legends), or explain why this information doesn’t apply to your submission:

We did not compute a sample-size beforehand. The observed differences were large and significant, we did not observe strong outliers in the data-sets, and the distributions were unimodal and only mildly skewed. Based on the central limit theorem we decided that a sample-size of >30 should be sufficient for our analysis.

**Replicates**

- You should report how often each experiment was performed
- You should include a definition of biological versus technical replication
- The data obtained should be provided and sufficient information should be provided to indicate the number of independent biological and/or technical replicates
- If you encountered any outliers, you should describe how these were handled
- Criteria for exclusion/inclusion of data should be clearly stated
- High-throughput sequence data should be uploaded before submission, with a private link for reviewers provided (these are available from both GEO and ArrayExpress)

Please outline where this information can be found within the submission (e.g., sections or figure legends), or explain why this information doesn’t apply to your submission:

In each experiment, the cells were randomly selected. As the cells are genetically identical, data from biological replicates were pooled together in the graphs. In the screen of nup-localization toward the DNA cluster we tested different proteins/reporters of the same class (e.g. Nup60, Mlp1,Mlp2 and Nup170, Nup133 in Fig. 1, 2, 5, 7), tested similar knockouts (Fob1, Sgf73 in Fig. 3), we tested different references (Nup159, Nup84) or different approaches (Fig. 1 vs Fig. 2). This is indicated in the figure legends. No data was excluded from the analysis.

**Statistical reporting**

- Statistical analysis methods should be described and justified
- Raw data should be presented in figures whenever informative to do so (typically when N per group is less than 10)
- For each experiment, you should identify the statistical tests used, exact values of N, definitions of center, methods of multiple test correction, and dispersion and precision measures (e.g., mean, median, SD, SEM, confidence intervals; and, for the major substantive results, a measure of effect size (e.g., Pearson's r, Cohen's d)
- Report exact p-values wherever possible alongside the summary statistics and 95% confidence intervals. These should be reported for all key questions and not only when the p-value is less than 0.05.

Please outline where this information can be found within the submission (e.g., sections or figure legends), or explain why this information doesn’t apply to your submission:

(For large datasets, or papers with a very large number of statistical tests, you may upload a single table file with tests, Ns, etc., with reference to sections in the manuscript.)

The statistical analysis methods are reported in the figure legends and material and method section. The sample sizes and p-values are always indicated, as well as the distribution of the data in a standard box-plot representation.

**Group allocation**

- Indicate how samples were allocated into experimental groups (in the case of clinical studies, please specify allocation to treatment method); if randomization was used, please also state if restricted randomization was applied
- Indicate if masking was used during group allocation, data collection and/or data analysis

Please outline where this information can be found within the submission (e.g., sections or figure legends), or explain why this information doesn’t apply to your submission:

The single cells were randomly selected from a large pool of cells in Fig. 4. For the other figures was a random field of view blindly selected, and all relevant cells from the field of view were analysed. The criteria for identifying the relevant cells are described in the method section.

**Additional data files (“source data”)**

- We encourage you to upload relevant additional data files, such as numerical data that are represented as a graph in a figure, or as a summary table
- Where provided, these should be in the most useful format, and they can be uploaded as “Source data” files linked to a main figure or table
- Include model definition files including the full list of parameters used
- Include code used for data analysis (e.g., R, MatLab)
- Avoid stating that data files are “available upon request”

Please indicate the figures or tables for which source data files have been provided:

The original source data is included for all the data figures in the manuscript. A table with the strains and plasmids used in this study is included as well.
